# Spray Deposition Synthesis of Locally Ordered Mesoporous Polycrystalline Titania Films at Low Temperature

**DOI:** 10.3390/molecules27010303

**Published:** 2022-01-04

**Authors:** Gunnar Símonarson, Antiope Lotsari, Anders E. C. Palmqvist

**Affiliations:** Department of Chemistry and Chemical Engineering, Chalmers University of Technology, 412 96 Göteborg, Sweden; gunnarsim@gmail.com (G.S.); alotsari@gmail.com (A.L.)

**Keywords:** spray coating, mesoporous, titania, TiO_2_, evaporation-induced self-assembly

## Abstract

A low-temperature spray deposition synthesis was developed to prepare locally hexagonally ordered mesoporous titania films with polycrystalline anatase pore walls in an evaporation-induced self-assembly process. The titania film preparation procedure is conducted completely at temperatures below 50 °C. The effects of spray time, film thickness, synthesis time prior to spray deposition, and aging time at high relative humidity after deposition on the atomic arrangement and the mesoorder of the mesoporous titania were studied. We find the crystallite size to depend on both the synthesis time and aging time of the films, where longer times result in larger crystallites. Using the photocatalytic activity of titania, the structure-directing agent is removed with UV radiation at 43–46 °C. The capability of the prepared films to remove the polymer template increased with longer synthesis and aging times due to the increased crystallinity, which increases the photocatalytic efficiency of the titania films. However, with increasingly longer times, the crystallites grow too large for the mesoorder of the pores to be maintained. This work shows that a scalable spray coating method can be used to prepare locally ordered mesoporous polycrystalline titania films by judiciously tuning the synthesis parameters.

## 1. Introduction

Since Fujishima and Honda discovered the photoelectrolysis of water using titania in 1972 [1], a continuous interest from the research community as well as the industry has generated a wealth of knowledge about the synthesis and properties of titania. There are several structural parameters that affect the properties and performance of titania, including the degree of crystallinity, the polymorph type, and the structure on the nanometer scale. Mesoporous titania, with a pore size of 2–50 nm, has attracted increasing research interests, offering high surface area, controllable pore size, and attractive and tunable properties for applications such as the conversion of solar energy to electrical energy [2], for removal of organic pollutants in water and air [3], and as an electrode material in lithium ion batteries [4].

Ordered mesoporous titania is typically prepared in a multi-step process where the structure is achieved by co-assembly of the titania precursor and surfactant molecules, which act as templates to achieve the desired structure [5,6,7,8]. In general, ordered mesostructures of titania are challenging to achieve due to the high reactivity of the titania precursor. However, mesoporous titania films are commonly prepared by evaporation-induced self-assembly (EISA). In the EISA approach, the precursor and the surfactant are dissolved in low concentrations in an ethanol/water solution [9]. Subsequently, the solvents are evaporated, leading to progressively higher concentration of the surfactant, which induces the formation of ordered liquid crystalline phases and co-assembly of the surfactant and the titania precursor. Subsequently, the structure is consolidated by aging in a humid environment and the template was removed typically by solvent extraction or calcination at high temperatures [10,11]. To induce the co-assembly of the surfactant and the titania precursor, the solvent evaporation is typically achieved with deposition techniques such as spin coating or dip coating [3]. A drawback of these coating methods is that they are not easily scalable to large surfaces.

Another much less explored technique is spray deposition to induce solvent evaporation and the formation of ordered mesostructured titania films. By using spray deposition, potentially very large surfaces and curved substrates may be coated. In the literature, there are a few reports on spray deposition to prepare crystalline mesoporous titania films with some success [12,13,14,15]. The morphology on the mesoscale of the spray coated films has been characterized in situ with GISAXS [14], the effect of adding crystalline preformed crystalline titania particles was studied [15], and films with tunable pore size have been prepared [13]. In these reports, the substrate was heated to 50–80 °C during the spray deposition, and further heat treatment at 500 °C was conducted to remove the template. In addition to removing the template, the high-temperature treatment increases the crystallinity and titania crystallite size [3]. Increasing the crystallinity is beneficial particularly for photocatalytic and photovoltaic applications. However, the high-temperature treatment has a detrimental effect on the ordered porous structure, which is usually attributed to the growth of the crystals from the pore walls into the pores [10]. With the aim of maintaining the ordered mesostructure and obtaining a degree of crystallinity, there is a large interest in developing low-temperature synthesis methods. 

There are several reports of mesoporous titania with small crystals prepared at low temperatures; however, these reports either lack evidence for order in the mesoporous structure or offer very limited crystallinity [16,17,18,19,20]. In 2011, Nilsson et al. reported a synthesis of crystalline hexagonally ordered mesoporous titania films carried out at low temperatures (ca. 40 °C) [21]. The films were prepared by spin coating a dilute solution of the Pluronic™ F127 triblock copolymer and titania precursor; subsequently, the films were aged at 75% relative humidity (RH), and finally, the polymer template was removed with UV radiation. Further studies on the system developed by Nilsson et al. have shown that longer synthesis times before spin coating results in increased crystallinity and a more disordered mesostructured [22], aging at high RH is essential to achieve an ordered mesostructured [23], and the polymorph selectivity is different in the synthesis solution prior to deposition than it is during aging of the films [24].

**Figure 1:**
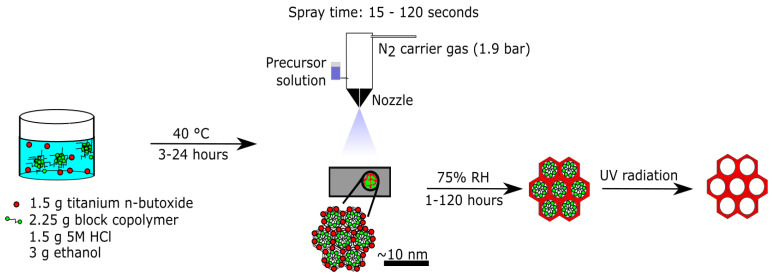
Schematic illustration of the spray deposition synthesis of mesoporous titania films.

In the present study, we report the development of a scalable spray deposition synthesis to prepare locally ordered mesoporous titania films, which was carried out completely at temperatures below 50 °C. The reaction solution is prepared at 40 °C, the solution is sprayed onto the substrate without any heating, and the template is removed by UV treatment at temperatures below 50 °C. This novel synthesis procedure is illustrated schematically in Figure 1. To the best of our knowledge, this is the first study that reports a spray deposited locally ordered mesoporous crystalline titania film completely prepared in this low-temperature range. The procedure developed here provides control over the film thickness and the crystallinity. Furthermore, this procedure may be used to prepare locally ordered mesoporous titania films over large areas and on heat-sensitive and curved substrates. Moreover, we expect the method can be extended to other materials and offers a way to prepare films of mesoordered silica and other oxides.

## 2. Results and Discussion

Figure 1 illustrates graphically the spray deposition synthesis developed here. The synthesis procedure can be summarized in four steps: (i) the Pluronic™ F127 block copolymer is dissolved in a solution of ethanol and HCl, and the titania precursor is subsequently added to the mixture and it is stored at 40 °C for 3–24 h in a closed vessel; (ii) the solution is spray deposited on a substrate and the solvents are evaporated, inducing the formation of an ordered liquid-crystalline phase and the co-assembly of the polymer and the titania precursor; (iii) aging at high RH for 1–120 h induces further condensation and crystallization of the titania species; and (iv) removal of the polymer template by UV radiation. The spray deposition was carried out using a commercial Aztek A470 Airbrush with a 0.30 mm general purpose nozzle, and nitrogen (1.9 bar) was used as a carrier gas. The nozzle was placed perpendicular 15 cm above the substrate, continuous spraying was applied for 15–120 s, and the solution flow rate was 2.2 mL/minute. The synthesis procedure is described in detail in the experimental section. The effects of the synthesis time prior to the spray deposition, the film thickness, controlled by spray time, and the aging time at high RH on the mesoorder and atomic order are investigated with SAXS and TEM analysis. Furthermore, the spray deposition procedure was validated by comparing the spray deposited films to spin coated films, which were prepared according to the previously reported procedure [21].

### 2.1. Effect of Deposition Method on Mesoorder

To evaluate the spray deposition procedure and to compare it to the previously reported spin coating procedure, the film’s mesostructure was assessed with SAXS. In previous reports, polycrystalline locally hexagonally ordered mesoporous titania thin films were prepared by depositing films via spin coating a reverse micellar solution onto glass slides [21,25]. The formation of the ordered structure from the micellar solution is a result of the solvent evaporation during the deposition. Although the solvent evaporation rates were expected to be highly dependent on the deposition method, the solution composition that had been used previously to prepare spin coated films was initially tried to prepare the spray deposited films.

**Figure 2:**
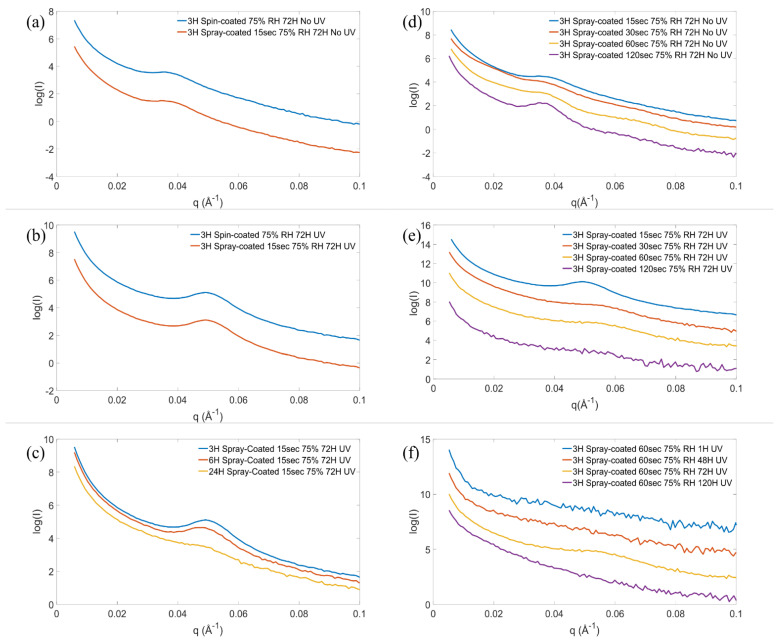
SAXS patterns of samples prepared with either spin coating or 15 s of spray coating from solution with 3 h synthesis time, before template removal (**a**) and after template removal (**b**). SAXS patterns of 15 s spray coated films prepared from solution with reaction times from 3 to 24 h, after template removal (**c**). Patterns of films with 15–120 s spray coated films before (**d**) and after template removal (**e**). SAXS patterns of 60 s spray coated films with aging time 1–120 h at 75% RH (**f**). The patterns are shifted in intensity for clarity.

Figure 2 shows the SAXS patterns of samples produced by the spray coating and the spin coating techniques, respectively, before template removal (a) and after template removal (b). For these samples, the reverse micellar solution was heated to 40 °C for 3 h prior to deposition and aged at high RH for 72 h after deposition. The spray time was 15 s for the spray coated films. Before template removal, both samples exhibit a main peak at a q-value of 0.036 Å^−1^. Using the photocatalytic activity of titania, the template was removed with UV radiation. The temperature in the sample position in the UV chamber was between 43 and 46 °C. After the template was removed, the peaks shift to a higher q-value of 0.049 Å^−1^, indicating a shrinkage of the structure. The d-spacing, *d*, was 17 nm and 13 nm prior to and after the template removal, respectively, for both methods of deposition. The shrinkage of mesoporous structures by calcination of the template has been reported before and was attributed to further condensation of the precursor during calcination [26]. Although the temperature reported here, during the UV treatment, was much lower than in a typical calcination, the UV treatment induces solvent evaporation, which promotes further condensation of the titania precursor. In addition to the peak shift, the peak intensity increased after the UV treatment due to a larger scattering contrast between titania and the empty pores as compared to titania and the polymer template. No additional higher-order peaks are observed in graphs (a) and (b), suggesting that the structure is locally ordered on the mesoscale. 

**Figure 3:**
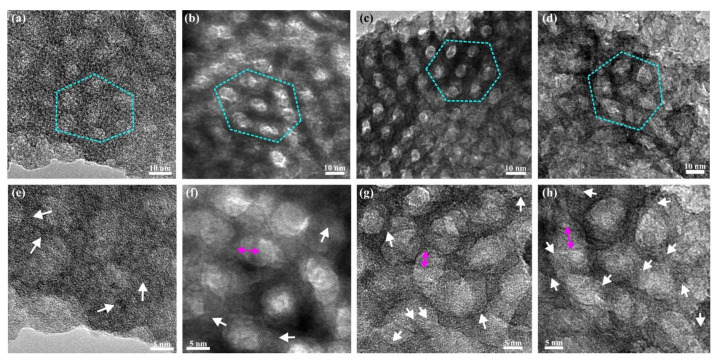
BF TEM images of mesostructured titania with synthesis time 3 h, before template removal (**a**), and after template removal with synthesis times of 3 h (**b**), 6 h (**c**), and 24 h (**d**). The hexagonal order of the pores has been outlined with cyan dotted lines. (**e**–**h**) HRTEM images of samples (**a**–**d**), respectively, in which some of the anatase crystalline areas are highlighted with white arrows. Magenta double arrows denote the shadowing of pores due to the inclined viewing direction.

To better evaluate the morphology and crystal structure of the mesostructured titania, HRTEM was employed. Figure 3a shows bright-field (BF) TEM micrograph of a sample prepared with 3 h synthesis time before template removal. At this point, filled with the polymer, the pore size is estimated to 6.5 ± 1 nm (Entry I, Table S1, Supplementary Materials), and the walls are of a similar thickness. Figure 3b shows a BF TEM micrograph of an identical sample after template removal, the pore size is estimated to 5.5 ± 1 nm (Entry II, Table S1, Supplementary Materials), and a hexagonal order is clearly observed in the mesoporous titania film. Figure 3f shows an HRTEM image of the same sample where the crystallinity is more clearly presented, and the d-spacing suggests the crystals are of the anatase polymorph. Similar results have been obtained by TEM analysis of the spin coated films [21]. Figure S1 (Supplementary Materials) shows an XRD pattern with broad peaks, further suggesting the presence of small crystallites in the structure. Due to the broad nature of the peaks, it was not possible to determine the crystalline phase type from the pattern.

The SAXS patterns and the TEM characterization illustrate that the spray deposited titania films are polycrystalline and possess a locally ordered mesostructure, demonstrating that the spray deposition procedure is a valuable method to prepare these films. In the following sections, the effects of the synthesis time at 40 °C, the aging time in high RH, and the film thickness are studied as means to further improve of the structural fidelity of the mesostructured films.

### 2.2. Effect of Synthesis Time on Mesoorder and Atomic Order

To study the effect of the synthesis time of the solution at 40 °C, prior to deposition, it was varied from 3 to 24 h, and the mesoorder was analyzed with SAXS measurements and TEM analysis. Figure 2c shows the SAXS patterns of titania films prepared from solutions with synthesis times of 3 and 6 h. The samples exhibit a similar degree of mesoorder while the sample with a synthesis time of 24 h has a less sharp and lower intensity diffraction peaks, suggesting that the material is less ordered. 

The BF TEM images in Figure 3a–d show the hexagonally ordered pores of the titania films. The images of films with 3 and 6 h synthesis time, as shown in Figure 3b,c, respectively, clearly show a local hexagonal mesoorder, but the sample prepared after 24 h synthesis time showed less order (Figure 3d). All samples exhibited the anatase polymorph, and the crystallites are better resolved after the template removal. This is depicted in the HRTEM images of Figure 3e–h in which white arrows indicate crystalline areas at the walls of the pores, which were determined to be the main crystalline phase in all samples. With increasing synthesis time, the crystals appear to have grown in size, and after 24 h synthesis time, the crystals have partly grown out of the walls and into the pores and distort the structure, as can be seen from the less spherical shape of the pores. This crystal growth presumably occurs by a further reaction of unreacted titania precursor species within the hydrophilic domains of the liquid crystalline template. The pore diameter and the crystallite sizes are listed in Table S1 in the Supplementary Materials.

### 2.3. Effect of Film Thickness on Mesoorder

The effect of film thickness, controlled by varying the spray time onto the glass slide, on the mesostructure was investigated by SAXS. The spray time was varied from 15 to 120 s, and the film thickness was 7–20 µm, as measured by a profilometer (Table S4 in Supplementary Materials). The SAXS patterns of the films before template removal are shown in Figure 2d and after template removal in Figure 2e. Before the template removal, the SAXS patterns all show a main peak at 0.036 Å^−1^, and the thicker films, with 30–120 s spray time, exhibit a second peak at 0.064 Å^−1^, indicating a higher degree of mesoorder in the thicker films. Increasing the thickness of the film decreases the solvent evaporation rate after deposition, which has been shown to improve the mesoorder [27]. The relative position of these peaks (1:√3) suggests that the structure is H1 hexagonal before the template removal [25]. However, only one peak is observed after the template removal, suggesting that the mesoorder is somewhat lost during the UV treatment. As expected, the peak is shifted to higher q-values for all samples after the UV treatment. Films prepared with spray time 30–120 s show a larger peak shift compared to the film with 15 s spray time, suggesting the mesostructure shrinks more in the thicker films. After the template was removed, it is observed that the peak features become less sharp with increasing film thickness, indicating that the locally ordered mesostructure is better maintained in the thinner films and the order becomes progressively more distorted with increasing film thickness after the template removal. As discussed in previous section, the structure shrinkage can be attributed to condensation of the titania precursor during the UV treatment and removal of the polymer template support. The further condensation is driven mainly by the increased titania precursor concentration due to evaporation of the solvents in the films during the UV treatment. The condensation of the thicker and thinner films is expected to proceed to a similar extent in this step, although the evaporation rate might be higher in the thinner film. However, the thicker films may be more dependent on the support of the polymer template, which may explain the more significant loss of mesoorder after the UV treatment.

### 2.4. Effect of Aging Time on Mesoorder and Atomic Order

**Figure 4:**
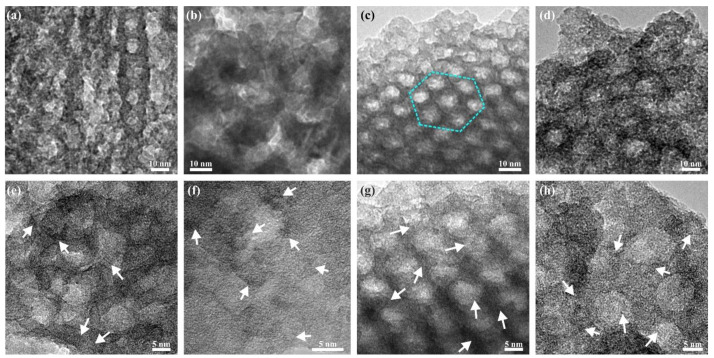
(HR)TEM images of mesoporous titania with 3 h synthesis time, deposited with 60 s spray coating and aged at 75% RH for (**a**,**e**) 1 h, (**b**,**f**) 48 h, (**c**,**g**) 72 h, and (**d**,**h**) 120 h. A hexagonal pore order was achieved for the sample aged for 72 h and is outlined with dotted cyan line (**c**). White arrows indicate some of the anatase crystallites.

The effect of varying the aging time of the as-deposited films at 75% RH from 1 to 120 h on the mesostructure and crystallinity of the films was studied. Figure 2f shows the SAXS patterns of the samples and indicates that an ordered mesostructure is only achieved for the sample aged for 72 h but not for samples aged for 1 h, 48 h, or 120 h. TEM images shown in Figure 4 confirm a clear local hexagonal order in the sample aged for 72 h (c), but no clear pore order was observed in the films aged for shorter or longer times, respectively. The HRTEM micrographs of Figure 4e–f show that films aged for 1 and 48 h appear to be mainly amorphous, but the pore walls also contain some small crystals of about 2 nm in size. These films turn orange in color after the UV treatment as opposed to white as the other samples, which suggests that the polymer template was not fully removed with the UV treatment. To quantify the amount of the polymer in the samples, combustion elemental analysis was used, and the carbon content was estimated to be 12% and 6.4% (*w*/*w*) for the samples aged for 1 h and 48 h, respectively. The UV template removal process relies on the photocatalytic activity of the material. Since amorphous titania, which was observed in the samples with shorter aging time, has a very limited photocatalytic activity, these samples have poor template removal capabilities. After aging for 72 h (g), the crystallinity in the film has increased, and the polymer template was removed upon UV illumination. The template is also removed for the sample aged for 120 h, but the ordered pore structure appears to be completely lost. The carbon content in the films is listed in Table S3, Supplementary Materials. Figure 4d,h show that the crystals have grown out of the pore walls, which appears to cause the loss of the ordered mesostructure. This confirms that the crystallites gradually grow during the aging time at 75% RH and that there is enough driving force, even at room temperature, for the crystal growth to disrupt the structure of the mesoporous titania. These results show that the aging time is a critical parameter for the atomic order of the film, which in turn strongly influences the nanometer-sized pores and the pore order of the film. The pore diameter and crystallite size of the films are listed in Table S2, Supplementary Materials. In a recent publication, where the electrochemical properties of the films prepared here were studied, it was shown that the mesoporous structures are retained after milling, mixing, and repeated electrochemical cycling [28]. 

## 3. Materials and Methods

### 3.1. Synthesis of Mesoporous Titania

Titanium n-butoxide (97%), Pluronic™ F127 (EO_100_PO_70_EO_100_) and hydrochloric acid (37%) were purchased from Sigma Aldrich, and ethanol (99.5%) was purchased from Solveco. The reaction solutions were prepared by stirring 3 g ethanol, 2.25 g F127 block copolymer, and 1.5 g 5 M HCl in a polypropylene bottle until the block copolymer was dissolved. When the polymer was completely dissolved, 1.5 g titanium butoxide was added dropwise to the solution. The mixture was stirred until the titanium butoxide was completely dissolved (approximately 10 min). The final molar composition of the reaction solution was (ethanol:F127:water:HCl:titanium n-butoxide) (65:0.18:69:6.9:4.4) mmol. The reaction solution was heated to 40 °C in a closed vessel in an oven and stored for 3–24 h. After the desired synthesis time, the reaction solution was deposited on glass slides (VWR 631–1551, 76 × 26 mm). Two deposition methods were used, spin coating and spray coating. The spin coated films were prepared by dripping 7 drops of reaction solution onto a glass slide while it was spun at 300 rpm for 5 s and subsequently at 1000 rpm for 10 s in a spinner (SPIN150 wafer spinner APT GmbH). The spray coated films were prepared using an A470 Airbrush, Aztek with a 0.30 mm general purpose nozzle (Aztek 9305 CX). The solution was placed in a cup holder coupled to the airbrush, and nitrogen (1.9 bar) was used as a carrier gas. The nozzle was placed perpendicular, 15 cm above the substrate, and continuous spraying was applied for 15–120 s at a flow rate of 2.2 mL/minute. After spin coating or spray coating, the deposited films were aged in a closed climate chamber at room temperature and 75% RH for 1–120 h. The RH was controlled with a saturated NaCl solution and monitored with a Testo hygrometer. After aging, the thin films were irradiated with UV light, wavelength λ = 254 nm and power density = 110 W/m^2^ (measured with an USB2000 Ocean optics^®^ spectrometer), in a closed chamber for 24 h to photocatalytically oxidize and remove the block copolymer. The temperature in the sample position in the UV chamber was 43–46 °C (measured with a thermocouple).

### 3.2. Characterization Techniques

Small Angle X-ray Scattering (SAXS) measurements were performed in a SAXSLAB Mat:Nordic SAXS/WAXS/GISAXS instrument with high-brilliance micro-focus CuK_α_ radiation source, wavelength λ = 1.54 Å and beam size 0.3 mm. A Pilatus 300 K detector was used to detect the scattered intensity and the sample to detector distance was 1084 mm. The samples were scraped off the substrate, gently ground and placed in a quartz capillary, 1.5 mm outer diameter (Hilgenberg), and measured for 450 s. The capillary and the detector are kept in vacuum to minimize air scattering. The d-spacing was calculated using the formula d = 2π/q. For structural characterization, conventional and high resolution (HR) transmission electron microscopy (TEM) were employed. The TEM samples were lightly ground in a mortar, dispersed in ethanol in a sonication bath, dropped onto a carbon-coated Cu grid and dried at room temperature. The experiments were performed on a Tecnai G2 microscope, operating at 200 kV. Combustion elemental analysis was performed with an Elementar vario MICRO cube to measure the residual carbon from the block copolymer. The sample was fully combusted in the presence of oxygen and the carbon content in the material was calculated from the CO_2_ released. The film thickness was measured with a KLA Tencor D100 profilometer. A groove was made in the film with a sharp object and the surface was scanned perpendicular to the groove with a sensor. The force applied was 0.03 mg and the scan speed was set to 0.07 mm s^−1^.

## 4. Conclusions

A spray deposition synthesis was successfully developed for the preparation of locally ordered mesoporous titania films with anatase polycrystalline pore walls. The benefits of spray deposition, compared to other deposition techniques, is the potential to coat a large surfaces and even curved substrates. Additionally, the low-temperature spray deposition method developed here can be used on thermally sensitive supports, as it is carried out completely below 50 °C. Furthermore, we show that the crystallinity and the film thickness can be controlled and illustrate the effects of synthesis time prior to deposition, aging time at high relative humidity after deposition, and the film thickness on the mesostructure and crystallinity. For synthesis times of 3 and 6 h, the mesoporous titania films show clear local hexagonal order, but after 24 h, the mesostructure becomes more distorted, and larger crystallites are observed. The crystal growth and eventual loss of the ordered mesostructure is also observed for films aged at high RH for up to 120 h. With 1 h and 48 h aging times, the titania is present predominantly as an amorphous phase and does not exhibit sufficient photocatalytic activity to remove the polymer template upon UV radiation. However, a local hexagonal mesostructure was achieved after aging for 72 h where the increased crystallinity suffices to photocatalytically remove the template while the crystallites are still mostly small enough to be contained within the pore walls and the mesostructure is retained. We expect that this method can be used to prepare locally ordered mesoporous titania films over large areas and on heat-sensitive and curved substrates. Moreover, we presume that the procedure can be extended to other materials and offers a way to prepare films of mesoordered silica and other oxides.

## Data Availability

The data presented in this study are available on request from the corresponding author.

## Supplementary Materials

The following are available online, Figure S1: X-ray diffractogram of the sample prepared with 3 h synthesis time, 15 s spray time, 72 h aging time, and UV treated, Figure S2: Nitrogen adsorption–desorption isotherms of the sample prepared with 3 h synthesis time, Table S1: Average pore diameter and crystallite sizes of samples with synthesis time 3–24 h, 15 s spray deposition time, and aging time of 72 h at 75% relative humidity (RH), Table S2: Average pore diameter and crystallite sizes of samples with 3 h synthesis time, 60 s spray deposition time, and aging time 1–120 h at 75% RH, Table S3: Carbon content of samples with 3 h synthesis time and varying aging times and thicknesses after UV treatment and a sample that was not UV treated for reference, Table S4: The film thickness of samples with 15 to 60 s spray time, after UV treatment.

## Sample Availability

Samples of the compounds are not available from the authors.
