# Supplementary material for: Spray Deposition Synthesis of Locally Ordered Mesoporous Polycrystalline Titania Films at Low Temperature
Source: Molecules. 2022 Jan 4;27(1):303. doi: 10.3390/molecules27010303 (PMC8746936; doi:10.3390/molecules27010303)
Supplement: Supplementary file 1 [file molecules-27-00303-s001.zip › molecules-1358954-supplementary.pdf]

molecules

MDPI

Supplementary Information

Spray Deposition Synthesis of Locally Ordered Mesoporous Polycrystalline Titania Films at Low Temperature

Gunnar Símonarson, Antiope Lotsari and Anders E. C. Palmqvist *

Department of Chemistry and Chemical Engineering, Chalmers University of Technology, 412 96 Göteborg, Sweden; gunnarsim@gmail.com (G.S.); alotsari@gmail.com (A.L.)
* Correspondence: anders.palmqvist@chalmers.se

1. Pore diameter and crystallite size

The average pore diameter and crystallite size of the prepared films as estimated from TEM images are presented in Table S1 and Table S2.

Table S1. Average pore diameter and crystallite sizes of samples with synthesis time 3-24 hours, 15 seconds spray deposition time and aging time of 72 hours at 75% relative humidity (RH).

Entry	Synthesis tme [h]	UV treated	Pore diameter [nm]	Crystallite size [nm]
I	3	No	6.5 ± 1.2	2.7 ± 1.9
II	3	Yes	5.5 ± 1	3.6 ± 1.1
III	6	Yes	6.8 ± 1.1	3.8 ± 0.9
IV	24	Yes	8.5 ± 1.9	4.8 ± 1.1

Table S2. Average pore diameter and crystallite sizes of samples with 3 hours synthesis time, 60 seconds spray deposition time and aging time 1-120 hours at 75% RH. The samples were all UV treated.

Entry	Aging time [h]	Pore diameter [nm]	Crystal size [nm]
I	1	6.1 ± 1	2.3 ± 0.6
II	48	5.5 ± 1.3	2.5 ± 0.7
III	72	5.6 ± 0.7	3.6 ± 0.6
IV	120	9 ± 1.1	4.5 ± 0.9

2. Carbon content in samples after UV treatment

The carbon content of selected samples measured after UV treatment is listed in Table S3.

Table S3. Carbon content of samples with 3 hours synthesis time and varying aging times and thicknesses after UV treatment and a sample that was not UV treated for reference.

Entry	Spray time [s]	Aging time [h]	UV treated	Carbon content [w/w %]
I	60	1	No	44.9
II	60	1	Yes	12.0
III	60	48	Yes	6.4
IV	120	48	Yes	8.2
V	60	72	Yes	2.6
VI	60	120	Yes	1.5

3. Film thickness

The thickness of the UV treated films was measured with a KLA Tencor D100 profilometer and is listed in Table S4.

Table S4. The film thickness of samples with 15 to 60 seconds spray time, after UV-treatment.

Spray time [sec]	Thickness [µm]
15	7 ± 1
30	13 ± 2
60	20 ± 2

4. XRD patterns

XRD pattern of the sample prepared with 3 hours synthesis time, 72 hours aging time at high RH. Two very broad peaks are observed, suggesting very small crystallites. It was not possible to determine the phase composition due to the very broad peaks in the pattern.

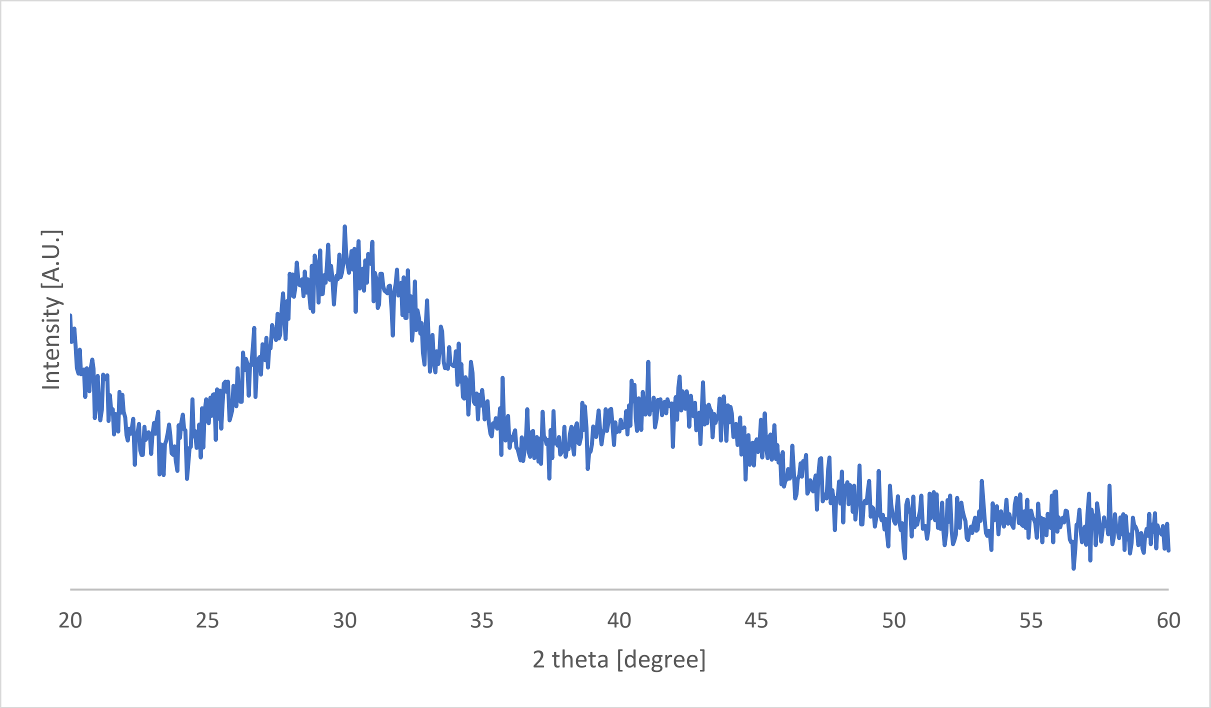

Figure S1. X-ray diffractogram of the sample prepared with 3 hours synthesis time, 15 seconds spray time, 72 hours aging time and UV treated.

5. Nitrogen sorption measurements

Nitrogen sorption measurements were performed on the sample prepared with 3 hours synthesis time and 72 hours aging time and the adsorption/desorption isotherm is presented in Figure S2. The BET surface area was calculated to 150 m^2 g^{-1}.

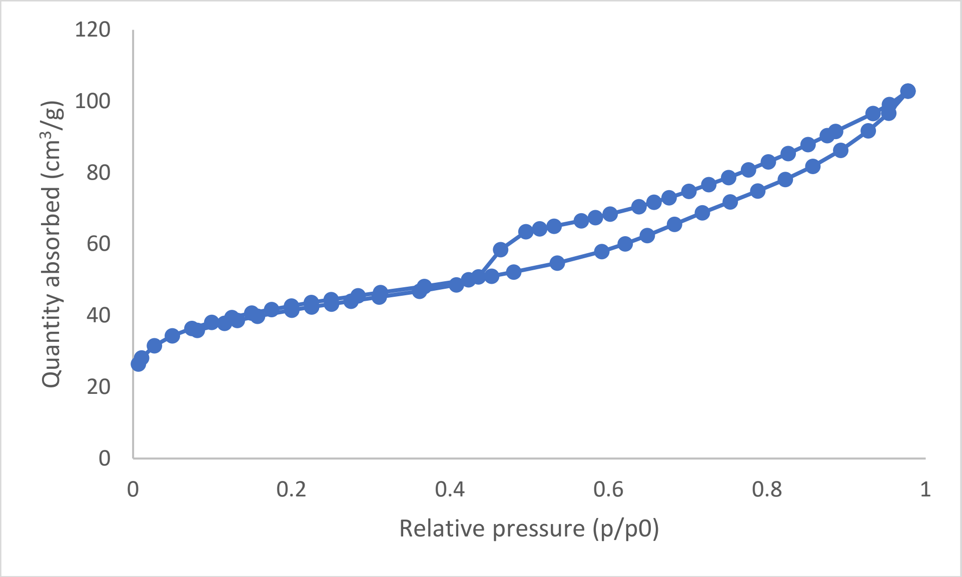

Figure S2. Nitrogen adsorption-desorption isotherms of the sample prepared with 3 hours synthesis time.
